# O9-5 Workplace sitting associated with self-rated perceived global health

**DOI:** 10.1093/eurpub/ckac094.069

**Published:** 2022-08-29

**Authors:** Lena V Kallings, Victoria Blom, Gunnar Andersson, Peter Wallin, Elin Ekblom-Bak

**Affiliations:** 1 Åstrand Laboratory of Work Physiology, The Swedish School of Sport and Health Sciences, GIH, Stockholm, Sweden; 2 Department of Sport and Health Sciences, The Swedish School of Sport and Health Sciences, GIH, Stockholm, Sweden; 3 Research Department, HPI Health Profile Institute, Danderyd, Sweden

**Keywords:** Total sitting, breaks, prolonged sitting, health risk, exercise

## Abstract

**Introduction:**

Total self-reported sitting time is associated with higher risk for cardiometabolic disease and mortality, while breaks in prolonged sitting has positive cardiometabolic effects. However, less is known about the associations of domain specific sitting and breaks at work and self-rated global health, likewise if physical activity could influence the associations.

**Methods:**

36,120 adults (42% women) from the Swedish working population who participated in a nationwide occupational health service screening 2014 -2018 were included in this cross-sectional study. Sitting duration and frequency of breaking sitting time at work, self-rated global health, exercise, leisure time sitting, diet, smoking and stress were self-reported. Cardiorespiratory fitness was estimated by a submaximal cycle test and BMI assessed through physical examination. Occupation was classified to requiring university competence or not. Logistic regression modelling assess OR (95% CI) associated between poor global health and decreased levels of workplace sitting and increased breaks in workplace sitting.

**Results:**

Having poor perceived global health was associated with increasing levels of workplace sitting, OR 0.65 (0.57-0.74) for sitting 75% of the time vs. sitting almost all time. Association were found between having poor perceived global health and lower frequency of breaking up workplace sitting every 30 minutes, in people sitting more than half of their working time, OR 0.60 (0.51-0.69) for occasionally vs. seldom breaking up sitting. The association were affected by sex, type of work, exercise habits, and sitting during leisure time. When sitting almost all the time OR for poor global health was 0.48 (0.39-0.60) for regular exerciser vs. no regular exercise. Within the no regular exercise group the OR 0.75 (0.63-0.89) for having poor global health was lower if not sitting almost all the time.

**Conclusion:**

Sitting almost all the time at work and not taking breaks, are associated with increased risk for perceived poor global health. The associations are affected by sex, type of job, exercise habits and sitting during leisure time. People who have to sit almost all their time at work, should be recommended to exercise on regular weekly bases and/or decrease their leisure time sitting to reduce the risk for poor health.

